# Exploring risk factors of drive for muscularity and muscle dysmorphia in male adolescents from a resource-limited setting in Burkina Faso

**DOI:** 10.1038/s41598-023-46863-w

**Published:** 2023-11-17

**Authors:** Valentin Terhoeven, Christoph Nikendei, Mamadou Bountogo, Hans-Christoph Friederich, Lucienne Ouermi, Ali Sié, Guy Harling, Till Bärnighausen

**Affiliations:** 1 Department of General Internal Medicine and Psychosomatics, Centre for Psychosocial Medicine, University Hospital Heidelberg, Thibautstrasse 4, 69115 Heidelberg, Germany; 2 Institute for Global Health, University College London, London, UK; 3 Heidelberg Institute of Global Health (HIGH), University of Heidelberg, Heidelberg, Germany; 4 Department of Global Health and Population, Harvard T.H. Chan School of Public Health, Boston, MA USA; 5 Africa Health Research Institute (AHRI), Durban, KwaZulu-Natal South Africa; 6 Centre de Recherche en Santé de Nouna, Nouna, Burkina Faso; 7 Department of Epidemiology & Harvard Center for Population and Development Studies, Harvard T.H. Chan School of Public Health, Boston, MA USA; 8 MRC/Wits Rural Public Health & Health Transitions Research Unit (Agincourt), University of the Witwatersrand, Johannesburg, Gauteng South Africa

**Keywords:** Epidemiology, Preventive medicine, Psychology and behaviour, Psychiatric disorders

## Abstract

In low-income countries, Muscle Dysmorphia (MD) has only been investigated in adult south African amateur-bodybuilders. To date, there is no epidemic study about MD or its cardinal symptom “drive for muscularity” (DFM) and its impact on young men’s lives in African low-income settings. We analyzed a population-representative cross-sectional study of 838 adolescent males aged 12–20 in the rural northwestern Burkina Faso. Participants were assessed for MD with the research criteria of Pope and its cardinal symptom DFM based on the DFM scale (DMS). Since DFM has not been studied in a comparable sample so far, all possible influencing variables were examined exploratively in a linear regression model. Many respondents were underweight (41.5%) and few overweight (1.3%). No-one met standard clinical MD criteria. While 60.1% of 837 wished to be more muscular, only 8.7% of 824 desired a lower body-fat percentage. Regression analysis revealed that higher DMS scores were associated with greater internalization of the muscular body ideal, going to school, living in a rural area, older age, and a history of having faced sexual harassment or assault, but not with media exposure. Our results show that levels of DMS in Burkinabe adolescents were elevated. Risk factors for DFM in environmental circumstances where undernutrition and poverty are common are discussed.

## Introduction

Muscle dysmorphia (MD) is characterized by a pathological preoccupation that one’s own body is insufficiently lean and muscular^1^. Affected people follow strict training and dieting regimes to gain muscle and lose fat, frequently using performance-enhancing substances; paradoxically, this occurs even when affected individuals are more muscular than the average^1^. Furthermore, MD related behaviours, like excessive training and the obsessive preoccupation with muscularity, leads to the neglect of other social, recreational or occupational activities, and continue despite knowledge of adverse physical and psychological effects^2^. The cardinal symptom of MD syndrome is drive for muscularity (DFM). DFM is defined as the desire for a muscular body, which is influenced by the internalization of a muscular body ideal^3^. DFM alone—unlike the mental disorder MD—is not always pathological, but is considered a risk factor for development of MD. Previous research has described DFM as a continuum of severity that ranges from the absence of interest in physical appearance to the point of self-damaging health behaviors similar to those seen with MD^4^. Research suggests that DFM might be a moderator of the association between frustrated basic needs and MD^5,6^. Furthermore, high DFM also predicts MD^7^.

Initially, the diagnostic classification of MD was linked to eating disorders. However, in 2015, MD was included in the Diagnostic and Statistical Manual of Mental Disorder, 5th edition (DSM-5) as a specific type of body dysmorphic disorder (BDD) within the chapter of obsessive–compulsive and related disorders^2^. There is evidence that MD leads to severe psychological suffering, reflected in psychiatric comorbidities such as depression, low quality of life, elevated substance abuse rates, and suicidality^8^. Research has also shown that BDD is often comorbid with depression^9,10^. Furthermore, males with BDD and additional MD, when compared to males with BDD without MD, showed a lower quality of life, were more likely to report a suicide attempt, and had a higher lifetime prevalence of substance abuse^11^. Most studies of MD have been conducted with weightlifters in higher-income countries in North America and Europe, finding prevalence rates varying from 1 to 54%^12^. One example is the Spanish sample (*N* = 734) of 562 male and 172 female bodybuilders, where a prevalence of 18.3% was found, and, regarding men with MD (*n* = 94), the use of anabolic androgenic steroids was reported by *n* = 12 (44.4%)^13^.

The knowledge about the etiology of MD is still limited and there is little progress in research^12^. Since MD is a disorder of the BDD spectrum, negative body image due to preoccupation with muscularity has come to be seen as a key issue. Studies in high-income countries suggest that an increase in the importance of muscular body ideals, most likely mediated by media^14,15^, has led to an increased sociocultural pressure to pursue a more muscular body. According to available literature, internalization of a muscular body ideal, being dissatisfied with one’s own body, and suffering from body distortion (i.e., misperceiving body features and body size) all strongly predict MD^16^. Most recently, a study tested a complete etiological model of eating disorders adapted to MD, which is applicable in low-income countries, for the first time^17^. The study underlined the relationship between social pressures to become both more muscular and leaner, DFM, and its mediation by the internalization of the ideal muscular body. The authors confirmed the major contribution of DFM in MD syndrome leading to an affective (i.e., negative affect) pathway and behavioral pathway (i.e., muscle-enhancing behaviors).

As age increases towards adolescence, there is an increased risk of experiencing body dissatisfaction or even body image disturbances^18^. On the one hand, media exposure among pre-adolescent boys in the rural United States has been found to be strongly associated with DFM^19^; similarly, French male adolescents with higher degree of internalization of media-provided body ideals experienced greater pressure to increase muscle and higher DFM^20^. On the other hand, negative social interactions in childhood and adolescence, such as victimization (e.g., sexual harassment and violence), bullying, and peer comparison, often result in a negative body image, potentially leading to a perceived lack of masculinity, which may be compensated for by a high DFM^12^. Regarding ‘sexual violence’, there is evidence that sexual violence/harassment in adolescent boys and girls has large effects on mental health, such as a higher risk of attempted suicide or a higher risk of psychological distress^21^. Moreover, a high proportion of individuals with BDD reported childhood abuse and neglect^22^. Sexual violence against Children in African adolescent populations seem to be a common and serious problem^23^. In line with this, a qualitative study conducted in England examined sociocultural and personal experiences leading to a high DFM in men^24^ and found that negative experiences and social interactions in childhood and adolescence (e.g., bullying and peer- and sibling comparison) resulted in negative self-image as well as feelings of perceived lack of masculinity. The perceived lack of masculinity might be compensated by a high DFM^24^. Similarly, there is also evidence for an unusually high number of rape victims among female weightlifters, which suggest compulsive weightlifting as a response to sexual assault^25^. Moreover, Gruber and Pope (1999)^25^ found that, in *N* = 75 women, compulsive weightlifting as well as drug abuse might be associated with a history of rape, defined as coercive sexual assault with vaginal penetration.

Another risk factor of MD includes body dissatisfaction, which is a result of the impact of societal norms. Societal norms are, in turn, structured by culture and ethnicity^26^. These may be shape body ideals and body image: there is evidence that boys from low-income countries are influenced by their families regarding appearance^27,28^ and that, particularly in LMICs, where Western media are less present, appearance pressures might be transmitted more by peers or family^29^. In contrast to current high-income country cultures, in traditional sub-Saharan (SSA) societies, a larger body size represents wealth, beauty, and the absence of illness^30^. Specifically, we worked in a setting in northwestern Burkina Faso where income and wealth levels are very low—median household annual expenditure is around US$450^31^—and many adolescents are not in formal education. Most residents, especially outside the town of Nouna, are employed either in small-scale farming—notably of corn, rice and cotton—or animal husbandry. Therefore, the nutritional status of adolescents in Burkina Faso makes it difficult to achieve the traditional African body ideal. DFM and/or the desire for a larger body weight stemming from the traditional African body ideal of beauty and health could therefore be reinforced as a motivator by the low weight. Regarding media exposure, recent socioeconomic transitions in SSA, including rapid urbanization, have been accompanied by greater exposure to high-income country media^32^. This media exposure in lower- and middle-income countries (LMICs) may lead to changes in body image (i.e., self-perception of muscularity and desired muscle) due to the adoption of high-income country beauty ideals^32^ and, thus, to an increasing DFM.

In the current study, we used theoretical framework based on the sociocultural model to investigate the pathways between appearance ideals, internalization, body dissatisfaction, and DFM. For this purpose, we have used the model from Rodger et al.^20^. The authors reviewed a model that sees *internalization and appearance comparison* as a central element that is influenced by pressure to gain muscle as well as to lose weight, ultimately leading to DFM and drive for thinness, which in turn lead to disordered eating. In detail, the Model propagates that the family, the peers, and the media (i.e., Tripartite Influence Model) convey a body ideal, so that sociocultural messages lead to internalization of a body image and behavior. If adolescents are taught typical traditional role models (e.g., masculinity in Burkina Faso), if stronger peers have more chances with girls, and, if the media (e.g., radio) reports about successful athletes, this can lead to a more muscular/athletic body ideal (internalization), which creates an internal pressure to put on weight—this ultimately creates a DFM. In line with previous research^33–35^, an aggregate media exposure variable was implemented, which included visual media, auditive communication media (i.e., radio), and muscular internalization. Due to the paradigm shift in 2015, we focused on the diagnosis of MD instead of eating behavior. Furthermore, since in Burkina Faso there is probably only a low and slowly beginning internalization of western values through e.g., media consumption, the model of Rodger et al.^20^ cannot be applied but only serves as the basis for possible influencing variables. Therefore, we decided to use an explorative approach to assess the emergence of DFM, the strongest predictor of MD.

MD is still understudied in SSA, despite researchers highlighting the importance of cross-cultural comparisons to gain knowledge on MD and its etiology^12^. One South African study showed that 15 out of 28 competitive adult body builders suffered from MD^36^, while another found a BDD prevalence of 5.1% in undergraduate students, similar to rates among high-income country student populations^37^. Even though pathological MD syndrome has only been proven/investigated in adult bodybuilders from African LMICs thus far, underlying BDD seems to be prevalent in adolescents from LMICs. In a cross-cultural study with adult male participants, Thornborrow et al. (2020)^29^ investigated body ideals and body image in LMICs (i.e., Uganda–Africa and Nicaragua–Central America) versus a high-income country (i.e., United Kingdome–Europe). The authors showed that Ugandan men had the lowest levels of DFM as compared with United Kingdom. Furthermore, they found that media exposure predicts the behavioral dimension of DFM, but, belonging to a Black African ethnic group, no association with higher DFM was reported. The study concluded that, due to cultural aspects, there are differences in body ideals between high- and low-income countries. However, within a socio-cultural model, pressures to gain muscle may be similar across cultures. Another study investigated satisfaction with muscularity as well as body fat in young adult male students from the Midwestern United States vs. Northeastern United States vs. Southwestern United States vs. Ukraine and mid-age (mean age = 37 years) Ghanaian (northwestern sub-Saharan Africa) participants^38^. The authors concluded that dissatisfaction with muscularity or body fat seem to be related with a traditional male role. However, only half of Ghanaian participants desired a more muscular body—which is less as compared to 90% in United States. In contrast, Ghanaian men perceived themselves as less muscular compared with other men and reported that women would prefer men with more muscular bodies. Interestingly, the study showed a significant correlation between higher BMI and higher self-perceived muscle mass, underlining the role of nutritional status as important variable regarding body dissatisfaction.

There is, however, very limited research among people in LMICs. Research on the topic of body image and related outcomes in Sub-Saharan Africa with men/adolescents is particularly sparse. Therefore, MD symptoms and its predictors in a rural West African adolescent male population are assessed for the first time in the present study. First, we evaluated whether male Burkinabè adolescents show the key feature of MD (i.e., DFM) as well as the internalization of a muscular- and lean body ideal before investigating how increasing media exposure influence potential MD risk factors. In an exploratory approach, we investigated whether (1) MD risk factors are prevalent, (2) known MD risk factors in high-income countries also play a pivotal role in this resource-limited setting, (3) adolescents who received more exposure to media show higher DMS scores and desire a lower percentage of body fat (i.e., lean body ideal). For this purpose, sociocultural models about MD and DFM from the existing literature^20^ were reviewed first in order to compile known associated variables as well as predictors of MD and DFM. Due to the exploratory approach and the fact that the current study is the first to investigate MD in a population-representative non-Western sample of adolescents, we used a regression model including more generously variables that could be shown to have an impact on DFM or MD.

## Methods

### Participants and procedures

We used baseline data from a cohort of adolescents aged 12–20 in rural Burkina Faso, part of the ARISE Adolescent Health Study^39,40^. The cohort sample was drawn from the Nouna Health and Demographic Surveillance System (HDSS) site overseen by the Centre de Recherche en Santé de Nouna (CRSN). The HDSS site, covering over 100.000 residents, comprises the market town of Nouna and 58 surrounding villages in the Boucle du Mouhoun province in western Burkina Faso^41^. CRSN conducts regular censuses of the resident population. Regarding ethnicity, participants were mainly Bwaba, Dafin, Mossi, Peuh, and Samo. To ensure representativity of the HDSS’s ethnic and urban makeup, the cohort was generated through a two-part stratified sampling procedure. First, 10 villages were purposively sampled to ensure that all main local ethnicities were included. Second, we drew a simple random sample of age-eligible adolescents from one of seven Nouna town sectors. For this study, we restricted our sample to male respondents. Baseline interviews were conducted in November and December 2017 at respondents’ homes in either French or a local language by 15 local interviewers. Interviewers were trained over three days using a study manual by three of the coauthors (MB, LO, GH) in French, with group practice using the materials and translating into key (largely unwritten) local languages. The final team of interviewers was selected based on a written test and mock interview observation. Once the study had begun, data was reviewed weekly for the first four weeks of data collection and interviewer-specific feedback provided where incongruities or likely misunderstandings were identified; the number of errors noted dropped rapidly as fieldwork progressed. Since local non-French languages are almost entirely oral in this area, it was not possible to prepare scripts for the study team to use when presenting questions to non-French speakers. However, as part of the training process, interviewers translated questions and response categories into other languages in small groups, agreeing on consistent wording to use within the team. The study collected self-reported information on socio-demographics, behaviours, health practices, and health outcomes. Interviews were conducted orally, with interviewers capturing responses using tablet computers. Interviews lasted 30–60 min with body image questions asked at approximately the mid-point. Ethical approval for this study was obtained from the Institutional Ethics Committee of the Centre de Recherche en Santé de Nouna and the Ethics Commission of the Heidelberg Medical Faculty exempted the study since only anonymized data were provided. All methods were performed in accordance with the guidelines laid down in the Declaration of Helsinki (most recent version: Fortaleza, Brazil, 2013). We also obtained approval from village elders, participants (written informed consent/assent), and parents/guardians (written informed consent if participant aged < 18).

### Measures

Weight and height measurements were measured twice each by fieldworkers; body-mass index (BMI) was calculated (BMI = kg/m^2^) based on the mean of each pair of measures; 19 BMI values were excluded for infeasible weight or height values. We categorized weight using the WHO BMI-for-age z-scores, considering those two standard deviations below the norm to be underweight and those ones with one standard deviation above to be overweight.

The MD research criteria proposed by Pope^1^ were used to determine MD syndrome. According to these criteria, in order to be diagnosed as having MD, a person must display the main criterion A (i.e., preoccupation of being insufficiently muscular), at least two out of four items of the B criteria which require that the MD syndrome cause clinically significant distress or impairment, and the criteria C (i.e., thinking the body is abnormal). See supplementary material (appendix p 4).

The Drive for Muscularity Scale (DMS) is a 15-item self-report scale which assess attitudes and behaviors related to preoccupation with being insufficiently muscular^42^. The DMS uses a 6-point Likert scale ranging from “never” (1) to “always” (6), with higher scores indicating greater drive for muscularity. The scale has been validated showing high internal reliability in English and in French for adolescent males in Canada and France^20,42,43^. With regard to similar study populations, Thornborrow et al. (2020)^29^ demonstrated good reliability in Uganda (Africa) as well as Nicaragua (Central America).

We measured internalization of media ideals using the internalization-general and internalization-athletic subscales of the Sociocultural Attitudes Towards Appearance Scale-3 (SATAQ-3)^44^, translated and validated in French^20,45^. The internalization-general subscale includes nine items and the internalization-athletic subscale five. All items use a 5-point Likert scale ranging from “never” (1) to “always” (5), with higher scores indicating higher levels of internalization^20^.

We assessed perceptual body image disturbance with Bodybuilder Image Grid-Original (BIG-O)^46,47^. The BIG-O is a two-dimensional figure rating scale designed to measure perceptual body image that consists of a grid of 30 male figures, which vary in leanness from “extremely low body fat” (1) to “extremely high body fat” (6) on the y-axis, and in muscularity from “extremely low muscle mass” (1) to “extremely high muscle mass” (5) on the x-axis. Respondents were asked to pick the figure that most closely represented their current figure and the figure they would ideally like to have, resulting in four different scores: (1) self-perceived muscle, (2) muscle ideal, (3) self-perceived body fat, (4) body fat ideal. Dissatisfaction for body fat and for muscle is calculated by taking the difference between respondents’ self-perceived and ideal column and row scores respectively (i.e., BIG-O Muscle dissatisfaction = ‘self-perceived muscle’ minus ‘muscle ideal’, BIG-O Body fat dissatisfaction = ‘self-perceived body fat’ minus ‘body fat ideal’. While positive BIG-O Muscle dissatisfaction scores reflect respondents’ desire of a more muscular body (i.e., muscular body image), negative BIG-O Body fat dissatisfaction scores reflect the desire of a lower percentage of body fat (i.e., leanness ideal).

Depression was measured using the six-item version of the Kutcher Adolescent Depression Scale (KADS-6), which has been validated in Canadian adolescents^48^. The KADS-6 uses a 4-point Likert scale (0–3; maximal score = 18) with a cutoff score of ≥ 6 as an indicator of possible depression. The KADS-6 was developed to diagnose depression in younger ages (i.e., adolescents) and evaluates low mood, feeling of worthlessness, feeling fatigued, loss of pleasure, feeling anxious as well as suicide temptations.

Quality of Life was measured with the Students’ Life Satisfaction Scale (SLSS), a seven-item self-report scale, which assesses global and context-free (i.e., domain-free and not evaluating specific a context such as school; e.g., “I have a good life” instead of “I have a good school life”) life satisfaction for adolescents, with higher scores on the SLSS indicating higher life satisfaction^49,50^. The items of the SLSS are as follows: (1) My life is going well, (2) My life is just right, (3) I would like to change many things in my life, (4) I wish I had a different kind of life, (5) I have a good life, (6) I have what I want in life, (7) My life is better than most of my age.

We measured ‘aggregate media exposure’ by summing frequency of use across four media: television, internet, magazines (each scaled as 0 for “never”, 1 for “some hours per month”, 2 for “some hours per week” and 3 for “some hours a day”), and radio (0 for “never”, 1 for “ < 1 h a day”, 2 for “1–2 h a day”; 3 for “ ≥ 2 h a day “), with a maximum possible value of 12^33^. See supplementary material (appendix p 5).

Finally, since sexual harassment and violence may affect body image and psychological wellbeing, we created a four-point scale based on self-reported lifetime experience of: verbal harassment; unwanted sexual touching; attempted rape; and rape. A maximum score of 4 could be achieved if all negative events are affirmed.

### Statistical analysis

First, we ran principal components analysis for DMS, to determine whether the high-income country factor structure was present in our SSA study sample, using varimax rotation; only items loading greater than .40 without cross-loading on any other factor above .40 were retained in the respective factor, as used elsewhere^19,43^. We then evaluated Cronbach’s alpha for all scales to estimate internal consistency. Next, after generating descriptive statistics, we ran bivariate regressions for DMS total score with all explanatory variables.

We conducted a multivariate linear regression to investigate whether known MD risk factors in high-income countries also predicted participants’ DMS sum score in this resource-limited setting. These included aggregate media exposure (continuous), going to school (binary), experience of sexual violence, age (12–13, 14–15, 16–17 and 18–20 years), dissatisfaction with muscle as well as body fat (muscular versus lean body ideal), internalization of body ideals, depression, and quality of life (all continuous). We also included the variable for village of residence (i.e., from Nouna town vs. village residence) in the regression analysis. Finally, as a sensitivity analysis, we re-ran the principal components analysis and our regression analyses excluding one interviewer who obtained substantially higher DMS values than all other interviewers (appendix p 1). Data were analysed using SPSS (Version 25; SPSS Inc., Chicago, IL., USA).

## Results

### MD criteria

A total of 948 of the 1268 adolescent males sampled (74.8%) were located and consented to participate. After the recruitment, we excluded 110 respondents from one outlier interviewer, resulting in a final sample size of *N* = 838. Mean and frequencies for all study variables characteristics are shown in Tables 1, 2. The majority of respondents met none of Pope’s three MD criteria (*n* = 590; 70.6%). While the presence of the criterion A found in this study sample did not cause clinically significant distress/impairment (criterion B), the main criterion A (i.e., preoccupation of being insufficiently muscular) was met by 29.4% (*n* = 246). Within the latter group, 20.7% (*n* = 51) also met criterion C (i.e., thinking the body is abnormal).

**Table 1:** Descriptive statistics for continuous variables.

	Possible range	*N* missing	Median (IQR)
Body ideal: SATAQ-3			
Total score	14 to 70	0	39 (30 to 51)
Internalization-general subscale	9 to 45	0	24 (16 to 30)
Internalization-athletic subscale	5 to 25	0	17 (13 to 21)
Media exposure: aggregate score	0 to 12	0	2 (1 to 3)
Dissatisfaction: BIG-O			
Self-perceived muscle	0 to 4	13	1 (0 to 2)
Self-perceived body fat	0 to 5	13	1 (0 to 2)
Desired muscle	0 to 4	6	2 (1 to 3)
Desired body fat	0 to 5	6	2 (1 to 3)
Muscle dissatisfaction	− 4 to 4	14	1 (0 to 2)
Body fat dissatisfaction	− 5 to 5	14	1 (0 to 2)
Drive for muscularity: DMS			
Total score	15 to 90	0	25.5 (18 to 33)
Size-related muscularity-oriented body image	4 to 24	0	9.5 (5 to 15)
Body part-specific muscularity-oriented body image	3 to 18	0	6 (3 to 9)
Preoccupation with weight lifting	4 to 24	0	4 (4 to 4)
Behavioural items relating to men becoming more muscular	3 to 18	0	3 (3 to 3)
Sexual harassment/violence	0 to 4	19	0 (0 to 0)
Quality of life: SLSS total score	7 to 42	2	21 (17 to 23)
BMI: WHO BMI-for-age z scores		21	− 1.14 (− 1.94 to − 0.43)

SATAQ-3, Sociocultural Attitudes Towards Appearance Scale-3; BIG-O, Bodybuilder Image Grid-Original; DMS, Drive for Muscularity Scale; BMI, body mass index; WHO, World Health Organization; SLSS, Students’ Life Satisfaction Scale; IQR, interquartile range.

**Table 2:** Descriptive statistics for categorical variables.

	*N* missing	*n* (%)
Depression: KADS total score (score ≥ 6)	0	10 (1.2%)
Resident in Nouna town	0	221 (26.4%)
Going to school	0	387 (46.2%)
Age (years)		
12–13	0	258 (30.8%)
14–15	0	204 (24.3%)
16–17	0	196 (23.4%)
18–20	0	180 (21.5%)
Categorization of Body Mass Index (WHO scale)		
Underweight-for-age (< − 2SD)	21	195 (23.3%)
Normal weight-for-age	21	603 (72.0%)
Overweight-for-age (> + 1SD)	21	19 (2.3%)

KADS, Kutcher Adolescent Depression Scale; WHO, World Health Organization.

### Principal components analysis

Cronbach’s alpha for the 15-item of the DMS was .86 and sampling adequacy was high (Kaiser–Meyer–Olkin = .84). We found four eigenvalues greater than one (Table 3; appendix p 3), accounting for 67.7% of all variance. In order, the factors consisted of: (1) size-related muscularity-oriented body image (4 items; *α* = .87); (2) body-part-specific muscularity-oriented body image (3 items; *α* = .97); (3) preoccupation with weight lifting (3 items; *α* = .63); and (4) behavioral items relating to men becoming more muscular (3 items; *α* = .71); Single item mean scores for Factor 1 and 2 were greater than those for Factor 3 and 4 (Table 3). Item 10 (“I think about taking anabolic steroids”) failed to load on one of the four factors.

**Table 3:** Rotated factor loadings for Drive for Muscularity Scale (*N* = 838).

	Mean	F1	F2	F3	F4
1. I wish I were more muscular	3.38	**.72**	.18	.12	.03
7. I think I would feel more confident if I had more muscle mass	2.34	**.83**	.25	.13	.11
9. I think I would look better if I gained 10 pounds	2.27	**.83**	.22	.16	.18
10. I think about taking anabolic steroids	1.10	.33	.04	− .08	.03
11. I think I would feel stronger if I gained a little more muscle mass	2.52	**.80**	.35	.11	.08
13. I think that my arms are not muscular enough	2.38	.31	**.92**	.06	.06
14. I think that my chest is not muscular enough	2.38	.29	**.93**	.04	.07
15. I think that my legs are not muscular enough	2.40	.29	**.92**	.09	.07
2. I lift weights to build more muscle	1.31	.20	.14	**.53**	.37
6. I feel guilty if I miss a weight-training session	1.05	.01	.06	**.80**	.10
8. Other people think I work out with weights too often	1.06	.06	.06	**.85**	− .04
12. I think that my weight-training schedule interferes with other aspects of my life	1.04	.03	− .01	**.72**	− .03
3. I use protein or energy supplements	1.24	.17	.08	.01	**.78**
4. I drink weight gain or protein shakes	1.10	.07	.01	.03	**.75**
5. I try to consume as many calories as I can in a day	1.19	.03	.05	.08	**.83**

Significant values are in [bold].

### BMI and psychometric assessment

**Figure 1:**
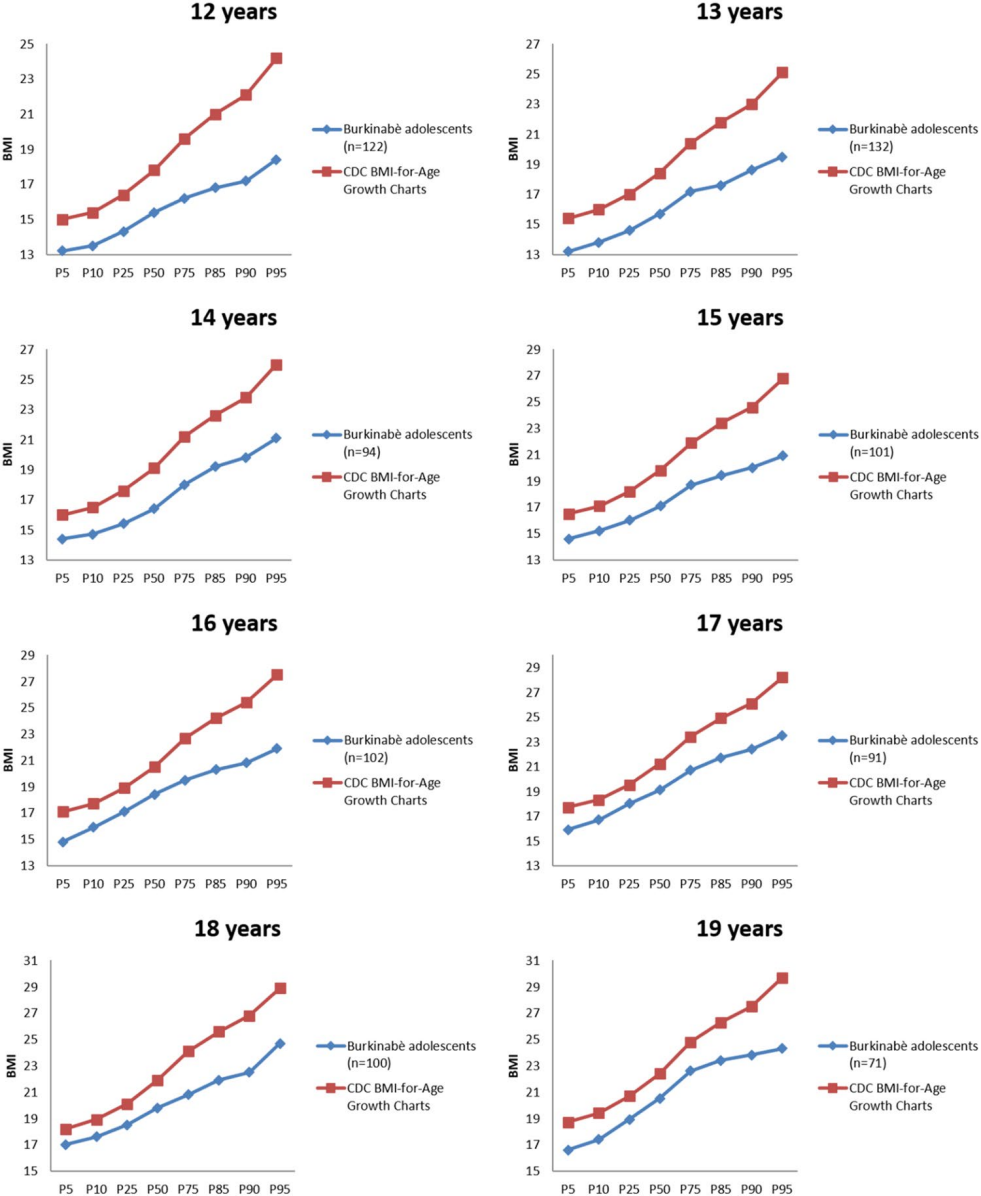
Comparison of BMI percentiles by age to the CDC BMI-for-Age Growth Charts for male adolescents aged 12–20.

BMI ranged from 10.0 to 27.7 kg/m^2^ (median = 17.6, IRQ = 15.8–19.7) and mean values by age were clearly below CDC growth chart levels (Fig. 1). While an expected number were normal weight (*n* = 460; 56.2%), based on the WHO Growth charts, 201 (24.5%) were of grade 1 thinness, 100 (12.2%) of grade 2 thinness, and 47 (5.7%) of grade 3 thinness. Only 11 respondents (1.3%) were overweight and none were obese. The BIG-O subscales ‘muscle dissatisfaction’ and ‘body fat dissatisfaction’ indicate that 524 out of 824 respondents (63.6%) desired to be more muscular, while 436 (52.9%) desired higher body fat percentage and only 72 (8.7%) desired lower body fat percentage. The SATAQ-3 was reliable (14 items; *α* = .91), as were both subscales (‘internalization-general’: *α* = .90; ‘internalization-athletic’ subscale: *α* = .81). Scores for the ‘internalization-general’ subscale were higher than those for the ‘internalization-athletic’ subscale.

With regard to psychosocial functioning, the KADS-6 depression scale was more reliable than the quality of life SLSS (*α* = .73, and *α* = .40, respectively). Only 1.2% of respondents had KADS-6 scores sufficient to be classified as possibly being depressed, while SLSS was normally distributed.

### Regression analyses

We analysed predicting factors for the DMS. In bivariate analyses, we found that greater internalization of the (1) athletic and (2) general muscular-oriented body ideal, (3) going to school, (4) older age, (5) living in areas that are more rural, (6) lower percentage of ideal body fat, and (vii.) a history of sexual harassment or assault were associated with higher DMS scores. The correlation matrix is shown in Table 4.

**Table 4:** Correlation Matrix of risk factors for drive for muscularity in Burkinabe male adolescents.

	1	2	3	4	5	6	7	8	9	10	11	12	13	14
School														
Nouna	− .228**													
BMI	− .056	− .095**												
12–14 years	.160**	.066*	− .243**											
14–15 years	.049	− .004	− .093**	− .349**										
16–17 years	− .059	.016	.085*	− .339**	− .318**									
18–20 years	− .170**	− .080**	.280**	− .349**	− .327**	− .318**								
SATAQ-3-G	.011	− .018	− .018	.047	− .065	.018	− .003							
SATAQ-3-A	.093**	− .046	.009	.002	− .029	.051	− .024	.624**						
Media exposure	.056	− .201**	.139*	− .220**	.003	.077*	.164**	− .015	.008					
BIG-O-M	− .010	− .029	− .111**	.046	.010	− .021	− .041	.138**	.138**	.021				
BIG-O-F	− .033	.010	− .110**	.099**	.048	− .072*	− .087*	− .046	− .044	− .090**	− .186**			
Abuse	− .073*	− .092**	.161**	− .178**	− .083*	.079*	.203**	.114**	.091**	.086*	− .095**	− .042		
KADS	.053	.016	− .001	.070*	.014	− .035	− .057	− .037	− .059	− .076*	− .057	.004	− .018	
SLSS	.063	− .095**	− .040	.019	.040	.002	− .065	− .055	− .086*	− .077*	− .120**	− .012	.006	.022

Pearson Coefficients with significant correlations at *p < .05, **p < .01.

DFM scale, Drive for Muscularity Scale; SATAQ-3, Sociocultural Attitudes Towards Appearance Scale-3; BIG-O, Bodybuilder Image Grid-Original; WHO, World Health Organization; BMI, body mass index; KADS, Kutcher Adolescent Depression Scale; SLSS, Students’ Life Satisfaction Scale.

In the omnibus multivariable regression, greater internalization of the athletic body ideal, going to school, older age, history of sexual harassment violence, and living in areas more rural remained significant, *R*2 = .19, *F*(12, 777) = 14.86, *p* < .001 (see Table 5). Results of risk factors for drive for muscularity in Burkinabe male adolescents, including the outlier interviewer, are shown in supplementary material (appendix p 2).

**Table 5:** Risk factors for drive for muscularity in Burkinabe male adolescents.

	*N*	Bivariate analyses*	Multivariate analysis†
Individual characteristics			
Going to school	837	1.53 (0.15, 2.90)	2.75 (1.36, 4.15)***
Nouna town vs. village residence	837	1.39 (− 0.16, 2.95)	2.95 (1.38, 4.51)***
WHO BMI-for-age z-scores	810	0.56 (− 0.08, 1.20)	0.19 (− 0.45, 0.83)
12–13 years	837	1	1
14–15 years	837	0.56 (− 1.29, 2.41)	0.66 (− 1.14, 2.45)
16–17 years	837	2.71 (0.84, 4.58)	2.58 (0.69, 4.47)**
18–20 years	837	3.35 (1.43, 5.26)	4.15 (2.11, 6.19)***
Muscle dysmorphia risk factors			
Body ideal: Internalization-general subscale (SATAQ-3)	837	0.22 (0.14, 0.29)	− 0.07 (− 0.16, 0.03)
Body ideal: Internalization-athletic subscale (SATAQ-3)	837	0.68 (0.57, 0.80)	0.71 (0.55, 0.86)***
Aggregate media exposure	837	0.21 (− 0.26, 0.68)	− 0.15 (− 0.61, 0.32)
Body image disturbance: Muscle dissatisfaction (BIG-O)	823	0.31 (− 0.15, 0.76)	0.12 (− 0.33, 0.58)
Body image disturbance: Body fat dissatisfaction (BIG-O)	823	− 0.70 (− 1.16, − 0.24)	− 0.27 (− 0.72, 0.19)
Psychosocial characteristics			
Sexual harassment/violence	818	1.84 (0.85, 2.83)	1.20 (0.23, 2.17)**
KADS not depressed vs. depressed	837	− 2.65 (− 8.98, 3.67)	− 0.13 (− 5.94, 5.68)
SLSS quality of life score	835	− 0.06 (− 0.20, 0.09)	0.04 (− 0.09, 0.18)

Data are B (95% CI) for the associations between risk factors for drive for muscularity and DMS scores in Burkinabe adolescent boys (multivariate analysis were run with *n* = 777). DMS, Drive for Muscularity Scale; SATAQ-3, Sociocultural Attitudes Towards Appearance Scale-3; BIG-O, Bodybuilder Image Grid-Original; WHO, World Health Organization; BMI, body mass index; KADS, Kutcher Adolescent Depression Scale; SLSS, Students’ Life Satisfaction Scale; B, regression coefficient; CI, confidence interval. *Bivariate unadjusted associations. †Multivariate associations, adjusted for the effects of all risk factors. Significance level are *p < .05, **p < .01, ***p < .001.

## Discussion

The necessity of investigating MD as well as DFM in Burkina Faso arose from various observations. First, a substantial amount of remarks were made by public health colleagues working in African LMICs regarding the trend of muscle training. Within this context, it was observed that more and more improvised gyms were being created. These consisted of self-made equipment and the impression quickly arose that there was a link between the two phenomena. Thus, we decided to exploratively examine this impression. Second, eating disorders (e.g., anorexia nervosa), which were seen as a first world disease, are now known to be found in LMICs^51,52^. We investigated the clinical syndrome of MD with the Pope research criteria on the one hand, and MD symptoms and MD risk factors like body dissatisfaction or DFM (the cardinal symptom of MD measured by the DMS) on the other. As for example in eating disorders, such as anorexia nervosa, we can investigate persons at risk (e.g., restraint eater) with a normal and thus not anorexic BMI. Similarly, MD can be investigated if the BMI is too low, although the diagnosis may never be made. Apart from that: before disease onset (at risk), BMI is usually within the normal range or tends to be too low.

While none of Burkinabe male adolescents showed MD *syndrome* by meeting the Pope MD diagnostic criteria nor by an increased BMI, as all participants were underweight, we found risk factors of MD’s cardinal symptom DFM in the current study sample where are circumstances of lower BMI and higher poverty rates than in high-income countries. This means, that in resource-limited setting like Burkina Faso, MD risk factors increase, but in a different phenomenological way: a specific characteristic of our study sample is that—besides the desire to be more muscular—they don’t want to be leaner. In contrast, “normal” MD is about achieving an extremely low proportion of body fat by a muscular appearance. Although Burkinabè adolescents apparently correctly perceived their low BMI, their desire to be bigger seems to arise from unrealistic muscular body ideals that probably overlap with the adaptive wish of restoring one’s own body weight within the normal BMI range^26^.

To date, the DMS has been applied to high-income countries, but neither to LMICs nor to SSA samples. In contrast to the two-factor^43^ or the three-factor solution^19^, a four-factor solution seems to be appropriate in our SSA study sample. This variation suggests that the construct of DFM might be interpreted differently in LMICs compared to high-income countries. As in high-income countries, the muscularity-oriented body image explained the greatest amount of variance in the DMS. However, the items referring to factor 3 (i.e., preoccupation with weightlifting) and factor 4 (i.e., behavioral items relating to men becoming more muscular) as well as item 10 (i.e., I think about taking anabolic steroids) seem less relevant in this SSA adolescent study sample.

Potential MD symptoms like DFM, media ideals and dissatisfaction with muscularity were prevalent within our sample. While rural male West African adolescents displayed a slightly less pronounced DFM based on the DMS (mean score: 25.5) than high-income country (i.e., French) adolescents (mean score: 28.99), the internalization of a general muscle-oriented and athletic body ideal based on the SATAQ-3 was far more pronounced in our study sample (mean scores: 24 and 17, respectively) as compared to high-income countries adolescents (15.83 and 10.17, respectively)^20^. In contrast to our hypothesis and our previous research finding that media exposure predicts the prevalence of eating disorders (e.g., anorexia nervosa) in Burkinabè adolescent girls^32^, in this male study sample, the degree of exposure to media through magazines, radio, television or internet were neither associated with the internalization of media ideals nor with DMS severity. Nevertheless, respondents had internalized the athletic body ideal as well as muscular body image. The risk of developing MD presenting higher DFM was greatest in adolescents presenting the athletic body ideal, going to school, with an older age, who reported a greater history of sexual harassment or violence, and who lived in rural areas. With regard to school status and the lack of influence of media exposure, our finding suggests that we are dealing with effects triggered by the peer group leading to higher social pressure. Thus, clinicians should implement bystander interventions (i.e., sensitization of peers to help each other) to handle peer pressure and to prevent sexual harassment or violence. Societies in Burkina Faso are pro-natalists (i.e., children as meaning of life) and children are raised to follow traditional patriarchal roles of the “ideal” man and the “ideal” woman; men (here masculinity) are authority figures and women (here femininity) are mothers and respectful helpers to their husbands^53^. More often than not, fathers do not spend time with their children. In particular, sons in Burkina Faso lack a father figure to identify with^54^, which would be important for the development of masculinity. The concept of masculinity and the endorsement of gender roles seem to have a great impact in the sense that males with MD reported higher adherence to masculine norms than controls^55^. A lack of identification with a father figure, the society consisting of a large number of children (which means that the attention of the parents has to be shared), traditional ideals, poverty, and a low-stimulating environment (e.g., no financial means) could be the reason why young boys from Burkina Faso want to impress their peer group through appearance in terms of a higher DFM. Furthermore, in Burkina Faso strong stereotypes of masculinity exist which are deeply rooted in gender norms such as to financially support the girlfriend; due to the precarious financial situation (poverty), however, there are also limited opportunities for young adult men to date other women^56^. Similarly, a compensation for the lack of financial means also could take place through the focus on the body.

Although, in literature, MD is associated with severe psychiatric comorbidity and our findings emphasize prevalent MD risk factors in this low-income study sample, psychological suffering was not depicted as expected. Only 1.2% of respondents were possibly depressed, while quality of life was normally distributed. Nevertheless, our findings of an unattainable muscular body ideal possibly being associated with DFM indicate that Burkinabè male adolescents have internalized a negative body image (e.g., that they are not sufficiently big enough). To prevent future psychological suffering, psycho-educational interventions on body image will likley need to be developed.

Some limitations of our findings need to be considered. First, due to the cross-sectional study design, we are unable to determine temporal ordering of events and, thus, causal relationships. This is particularly important given that our data suggest a transition in terms of body image in this setting towards a more muscular body ideal and, potentially, towards more clinical MD; testing this hypothesis would require follow-up in the future. Second, care must be taken in generalizing our findings beyond the study setting. It would seem likely that our results are applicable to other low-income, low media-saturation settings in SSA and perhaps elsewhere, but this should be tested empirically rather than assumed. Third, with regard to the validity of the BIG-O, we did not have time/resources to collect verbal feedback with regard to if participants struggled to pick a body from these images to accurately represent the kind of body they would like to have. Thus, results generated from using the BIG-O to assess body perceptions should be interpreted with caution. Therefore, future research should use other instruments in order to measure body perception or collect verbal feedback on the BIG-O. Another limitation is that, despite the carefully conducted training, there was an outlier interviewer, which significantly skewed the results in the initial evaluation. In future investigations, it makes sense to perform better real-time data checking.

In conclusion, media does not to be a determinant of increased DFM in this study sample. Nevertheless, Burkinabè adolescents show internalization of an unrealistic muscle ideal, which in turn is associated with DFM and may lead to MD in the future. In terms of a biopsychosocial understanding, our results highlight new research directions and have implications for public health research and interventions. Nutritional support and body image related psychoeducational interventions might be useful to support young men from Burkina Faso in order to restore weight within the normal BMI range and to acquire a more realistic body ideal.

### Supplementary Information

Supplementary Information.

## Data Availability

The dataset analysed during the current study is available from the corresponding author on reasonable request.

## Abbreviations

BDD: Body Dysmorphic Disorder
DFM: Drive for Muscularity (concept)
DMS: Drive for Muscularity scale (questionnaire)
LMICs: Lower- and middle- Income countries
MD: Muscle Dysmorphia
