# Supplementary material for: Exploring risk factors of drive for muscularity and muscle dysmorphia in male adolescents from a resource-limited setting in Burkina Faso
Source: Sci Rep. 2023 Nov 17;13:20140. doi: 10.1038/s41598-023-46863-w (PMC10656447; doi:10.1038/s41598-023-46863-w)
Supplement: Supplementary file 1 — Supplementary Information. [file 41598_2023_46863_MOESM1_ESM.docx]

# SUPPLEMENTARY TABLES

**Table S1. Interviewer’s responses for DFM scale scores comparing the outlier interviewer with the overall population**

|  | **Outlier**  **(*n*=110)** | **Without outlier**  **(*n*=838)** |
| --- | --- | --- |
| **Item 01.** I wish I were more muscular | 3·77 | 3·38 |
| **Item 07.** I think I would feel more confident if I had more muscle mass | 3·63 | 2·34 |
| **Item 09.** I think I would look better if I gained 10 pounds | 3·44 | 2·27 |
| **Item 10.** I think about taking anabolic steroids | 3·45 | 1·10 |
| **Item 11.** I think I would feel stronger if I gained a little more muscle mass | 3·60 | 2·52 |
| **Item 13.** I think that my arms are not muscular enough | 3·56 | 2·38 |
| **Item 14.** I think that my chest is not muscular enough | 3·52 | 2·38 |
| **Item 15.** I think that my legs are not muscular enough | 3·55 | 2·40 |
| **Item 02.** I lift weights to build more muscle | 3·63 | 1·31 |
| **Item 06.** I feel guilty if I miss a weight-training session | 1·47 | 1·05 |
| **Item 08.** Other people think I work out with weights too often | 1·41 | 1·06 |
| **Item 12.** I think that my weight-training schedule interferes with other aspects of my life | 1·44 | 1·04 |
| **Item 03.** I use protein or energy supplements | 3·59 | 1·24 |
| **Item 04.** I drink weight gain or protein shakes | 3·39 | 1·10 |
| **Item 05.** I try to consume as many calories as I can in a day | 3·60 | 1·19 |
| **DFM scale sum score** | 47·05 | 26·72 |
| Data are single item mean scores of the outlier interviewer vs. all respondents without the outlier interviewer. | | |

**Table S2: Risk factors for drive for muscularity in Burkinabe male adolescents including the outlier interviewer**

|  | **N** | **Bivariate analyses*** | **Multivariate analysis†** |
| --- | --- | --- | --- |
| **Individual characteristics** |  |  |  |
| Currently not in vs in school | 947 | 2·04 [0·39, 3·70] | 2·44 [0·89, 3·99] |
| Nouna town vs. village residence | 947 | 0·36 [-1·53, 2·25] | 3·17 [1·44, 4·91] |
| WHO BMI-for-age z-scores | 913 | 0·40 [-0·37, 1·16] | -0·03 [-0·74, 0·68] |
| 12-13 years | 947 | 1 | -0·41 [-2·41, 1·59] |
| 14-15 years | 947 | 1·06 [-1·15, 3·27] | 1 |
| 16-17 years | 947 | 3·03 [0·77, 5·29] | 1·86 [-0·31, 4·02] |
| 18-20 years | 947 | 5·13 [2·81, 7·44] | 4·72 [2·42, 7·02] |
| **Muscle dysmorphia risk factors** |  |  |  |
| Body ideal: Internalization-general SATAQ-3 subscale | 947 | 0·31 [0·23, 0·39] | -0·03 [-0·12, 0·06] |
| Body ideal: Internalization-athletic SATAQ-3 subscale | 947 | 1·06 [0·93, 1·20] | 0·99 [0·82, 1·15] |
| Aggregate media exposure | 947 | 1·57 [1·13, 2·01] | 0·76 [0·33, 1·19] |
| Body image disturbance: Muscle dissatisfaction (BIG-O) | 931 | -0·66 [-1·20, -0·12] | -0·34 [-0·84, 0·17] |
| Body image disturbance: Body fat dissatisfaction (BOG-O) | 931 | -0·83 [-1·37, -0·30] | -0·19 [-0·68, 0·30] |
| **Psychosocial characteristics** |  |  |  |
| Sexual harassment/violence | 924 | 2·12 [0·99, 3·26] | 0·90 [-0·13, 1·93] |
| KADS not depressed vs. depressed | 947 | -1·52 [-8·92, 5·89] | 2·04 [-4·27, 8·35] |
| SLSS quality of life score | 945 | 0·60 [0·45, 0·74] | 0·49 [0·35, 0·63] |
| Data are B (95% CI) for the associations between risk factors for drive for muscularity and DFM scale scores in Burkinabe adolescent boys (n=874). DFM scale=Drive for Muscularity Scale. SATAQ-3=Sociocultural Attitudes Towards Appearance Scale-3. BIG-O=Bodybuilder Image Grid – Original. BMI=Body Mass Index. WHO=World Health Organization. KADS=Kutcher Adolescent Depression Scale. SLSS=Students’ Life Satisfaction Scale. B=regression coefficient. CI=Confidence Interval. *Bivariate unadjusted associations. †Multivariate associations, adjusted for the effects of all risk factors. | | | |

# Supplementary Figures

Figure S1: Factor Analysis Scree Plot


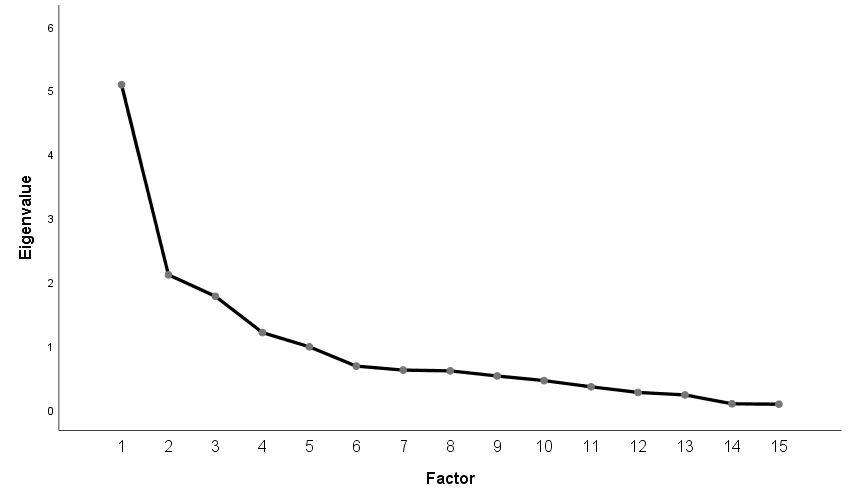


**Supplementary Interview**

**Pope Research Criteria**

1. **Criterion A:** Do you worry that your body is not slim and muscular enough?
   - No Skip to next scale
   - Yes
2. **Criterion B.1:** Do you frequently miss or leave social, work or recreational activities in response to a compulsive need to maintain the strict routine of physical or food activity.
   - No
   - Yes
3. **Criterion B.2:** Do you avoid situations in which your body is likely to be exposed, or do such situations make you distressed or anxious?
   - No
   - Yes
4. **Criterion B.3:** Bodily concerns are accompanied by clinically significant distress or social, occupational or other functional dysfunction.
   - No
   - Yes
5. **Criterion B.4:** You try to maintain physical activity, diet or substance intake to improve your physical performance, despite it having physical or mental effects
   - No
   - Yes
6. **Criterion C:** You are worried about becoming too small or insufficiently muscular and not to fear of gaining weight (anorexia) or being directed to another aspect of physical appearance (fear of Dysmorphism).
   - No
   - Yes

**Media Exposure**

1. Do you have access to a TV?
   - No
   - Yes
2. How often do you watch TV?
   - Never
   - Rarely (some hours per month)
   - Often (several hours per week)
   - Very frequently (several hours per day)
3. On average, how many hours a day do you spend viewing television?
   - _________ hours
4. What do you watch on TV?
   - Local programmes
   - International programmes
5. Do you have Internet access anywhere?
   - No
   - Yes
6. If Q5 == ‘Yes’: Do you have a smartphone with Internet access?
   - No
   - Yes
7. If Q5 == ‘Yes’: How often do you use the Internet?
   - Never
   - Rarely (some hours per month)
   - Often (several hours per week)
   - Very often (several hours per day)
8. If Q7 == ‘Often’ OR ‘Very Often’: How many hours a day do you spend on the Internet?
   - _________ hours
9. How often do you read magazines?
   - Never
   - Rarely (some hours per month)
   - Often (several hours per week)
   - Very often (several hours per day)
10. If Q9 ≠ ‘Never’: What kind of magazines do you read most often?
    - Local (Burkinabé)
    - International
11. How often do you have contact with people who are not from Burkina Faso?
    - Never
    - Rarely (some hours per month)
    - Often (several hours per week)
    - Very often (several hours per day)
12. How often do you have contact with people who are not from Africa?
    - Never
    - Rarely (some hours per month)
    - Often (several hours per week)
    - Very often (several hours per day)
13. Do you ever use social media or social networks?
    - No
    - Yes
14. Do you use any of the following social media?
    - Facebook
    - Twitter (X)
    - Instagram
    - Youtube
    - Reddit
